# Spleen EBV-positive inflammatory follicular dendritic cell sarcoma: a case report and literature review

**DOI:** 10.3389/fonc.2025.1640099

**Published:** 2025-08-22

**Authors:** Yi Xiao, Lanlan Li, Xiumei Zhan, Juner Xu, Yewu Chen, Qiuchan Zhao, Yinghao Fu, Xian Luo, Huadi Chen, Hao Xu

**Affiliations:** ^1^ Department of Hepatobiliary Surgery, Affiliated Hospital of Guangdong Medical University, Zhanjiang, Guangdong, China; ^2^ The First School of Clinical Medicine, Guangdong Medical University, Zhanjiang, Guangdong, China

**Keywords:** Epstein-Barr virus-positive inflammatory follicular dendritic cell sarcoma, spleen, inflammatory tumor, laparoscopic partial splenectomy, case report

## Abstract

Epstein–Barr virus-positive inflammatory follicular dendritic cell sarcoma (EBV+ IFDCS) is a rare tumor that typically arises in the liver or spleen and is characterized by spindle-shaped cells within a dense lymphoplasmacytic background. We report a case of a 64-year-old woman with an incidental splenic mass found during routine imaging. MRI showed a 4.8 cm lesion with progressive enhancement. The patient underwent laparoscopic partial splenectomy. Histopathological examination revealed features consistent with EBV+ IFDCS, including positivity for CD21, CD23, CD35, SMA, EMA, and EBER. EBV+ IFDCS often presents with nonspecific symptoms and imaging findings, making diagnosis challenging. Definitive diagnosis relies on histology, immunohistochemistry, and confirmation of EBV infection. Most cases follow an indolent clinical course and have a favorable prognosis after complete surgical resection, though rare aggressive cases have been reported. Understanding its clinicopathological and molecular features is essential for accurate diagnosis and management.

## Introduction

Follicular dendritic cell sarcoma (FDCS), first described by Juan Rosai in 1986, is a rare mesenchymal tumor originating from follicular dendritic cells (FDCs) of germinal centers (1). FDCS can be classified into classical FDCS and Epstein-Barr virus-positive inflammatory variant (EBV^+^ IFDCS) (2). The EBV^+^ IFDCS is a unique subtype associated with EBV infection, characterized by relatively indolent tumor behavior and distinctive pathological features. Histologically, it presents with spindle-shaped tumor cells admixed with numerous lymphocytes and plasma cells. Meanwhile, immunohistochemistry typically reveals expression of FDC markers and positive EBER *in situ* hybridization (3, 4). EBV^+^ IFDCS most commonly arises in the liver and spleen but has been rarely reported in the gastrointestinal tract, lung, and head and neck regions (5). Due to the lack of specific clinical and imaging features, preoperative diagnosis is difficult, and definitive diagnosis relies on histopathological examination (5). Herein, we present a case of EBV^+^ IFDCS arising in the spleen and conduct a comprehensive review of its clinicopathological features, molecular mechanisms, diagnostic criteria, and treatment strategies, aiming to improve recognition and inform clinical practice.

## Case presentation

**Figure 1:**
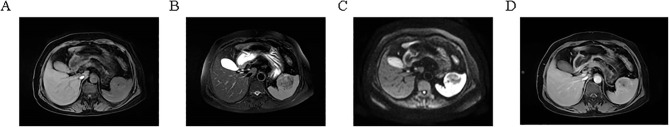
Magnetic resonance imaging of the splenic lesion. **(A)** T1-weighted image showing iso-intensity. **(B)** T2-weighted image showing slightly hypo-intense signal. **(C)** Diffusion-weighted image showing low to iso-signal intensity. **(D)** Contrast-enhanced image showing marked enhancement.

**Figure 2:**
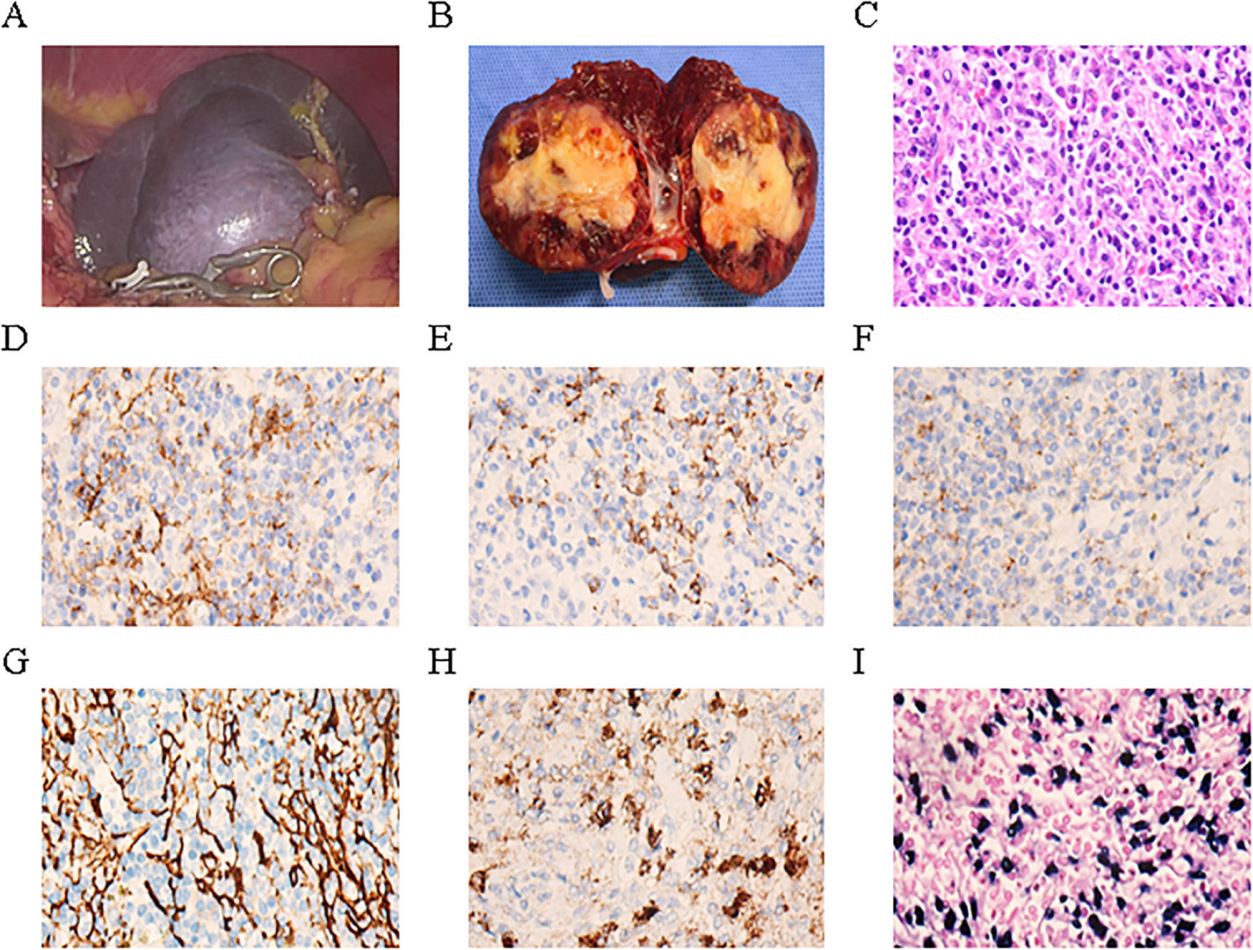
Epstein–Barr virus-positive inflammatory follicular dendritic cell sarcoma of the spleen in our case. **(A)** Intraoperative laparoscopic image of the mass. **(B)** Gross appearance of the tumor on cut section after resection. **(C)** Hematoxylin and eosin staining showing tumor histology. **(D–H)** Immunohistochemical staining demonstrates that the tumor cells express CD21 **(D)**, CD23 **(E)**, CD35 **(F)**, SMA **(G)**, and EMA **(H)**. **(I)** *In situ* hybridization reveals that the tumor cells are positive for Epstein–Barr virus -encoded mRNA.

A 64-year-old female presented to Guangdong Medical University Affiliated Hospital in April 2025 for evaluation of a splenic mass incidentally detected one month earlier during routine physical examination.The patient has no history of previous surgical procedures and has not received any vaccinations. The BMI is 23.72. Physical examination revealed no palpable mass below the left costal margin. MRI revealed a 4.7 × 4.8 × 4.8 cm lesion in the spleen, showing iso-intensity on T1-weighted images, slightly hypo-intense signal on T2-weighted images, and low to iso-signal intensity on diffusion-weighted imaging (**Figures 1A-C**). Contrast-enhanced imaging demonstrated a marked enhancement (**Figure 1D**), suggestive of sclerosing angiomatoid nodular transformation, a likely benign lesion. Preoperative blood tests and tumor markers AFP, CEA, CA19-9, and CA125 were all within normal limits. With a preoperative diagnosis of sclerosing angiomatoid nodular transformation, a partial splenectomy was performed to preserve the patient’s splenic immune function. Additionally, the patient expressed a desire to preserve the spleen, the patient underwent laparoscopic partial splenectomy on April 21, 2025. A drain was placed to monitor for bleeding and to ensure adequate drainage of residual intraperitoneal fluid. Intraoperatively, an about 5.0 cm mass was identified in the spleen, smooth-surfaced and poorly demarcated from surrounding tissue, soft in texture (**Figure 2A**). The mass in the mid-lower pole of the spleen was completely resected (**Figure 2B**). Histopathological analysis revealed EBV^+^ IFDCS with negative margins. Hematoxylin and eosin staining showed disrupted splenic architecture and scattered to dense spindle- to oval-shaped tumor cells amidst lymphoplasmacytic infiltrates (**Figure 2C**). Immunohistochemistry demonstrated positivity for CD21, CD23, CD35, SMA, and EMA (**Figures 2D-H**), and EBER *in situ* hybridization was positive (**Figure 2I**). The patient experienced no postoperative complications and was discharged uneventfully after drain removal. One month after surgery, the patient returned for follow-up. Platelet count was within normal limits, and coagulation function was normal.

## Discussion

Although EBV+ IFDCS is a malignant tumor, preservation of splenic function remains clinically important, especially in cases with localized disease and no evidence of systemic spread. The spleen plays a critical role in immune surveillance and hematologic regulation, and total splenectomy carries risks such as overwhelming post-splenectomy infection (OPSI), thrombosis, and long-term immune dysfunction (6, 7). Previous reports have demonstrated that partial splenectomy is feasible and safe in select patients, provided that negative surgical margins can be achieved (8, 9).

In the present case, we describe a rare instance of splenic EBV+ IFDCS successfully managed by laparoscopic partial splenectomy. This approach, guided by the tumor’s localized position in the lower splenic pole, benign imaging features, and the patient’s preference to preserve spleen function, contrasts with the more common practice of total splenectomy reported in the literature. Our case supports the use of spleen-preserving strategies in appropriately selected patients and contributes to expanding the surgical options for this rare entity.

To contextualize our findings and assess the current landscape of splenic EBV+ IFDCS, we conducted a comprehensive literature review. Specifically, we performed a search on PubMed up to July 15, 2025, using the following terms:(inflammatory follicular dendritic cell sarcoma OR inflammatory follicular dendritic cell tumor) AND (spleen OR splenic). This search yielded 45 publications, which were screened manually. We included only case reports or case series that provided sufficient clinicopathological details of EBV+ IFDCS located in the spleen. Articles were excluded if the tumor location was not splenic, if the diagnosis was unclear, or if pathological confirmation of EBV positivity was lacking.Based on these criteria, 8 cases (including the present case) were included in **Table 1**, allowing comparison of clinical features, imaging, treatment methods, and follow-up outcomes. This literature synthesis, along with our case, aims to improve recognition, guide individualized surgical decision-making, and enhance understanding of this rare disease.

**Table 1:** Clinical characteristics of spleen EBV-positive inflammatory follicular dendritic cell sarcoma.

Age/Gender	Clinical presentation	Imaging/Intraoperative findings	Treatment	Follow-up	Source
67/Female	Bleeding from the mouth, nose, and skin	CT scan indicated splenomegaly with a patchy low-density shadow measuring approximately 57*58 mm	Laparoscopic total splenectomy.	No tumor recurrence or metastasis was observed during the one-year follow-up	Jin J et.al (10)
59/Male	Bleeding gums and epistaxis	CT spleen mass and intrahepatic nodule, considering spleen tumor with intrahepatic metastasis	Splenectomy	No tumor recurrence or metastasis was observed	Leng DN et.al (11)
79/Female	Worsening abdominal pain, asthenia, and weight loss	CT was performed and showed a 70-mm splenic mass without any other radiological abnormality	Splenic biopsy and splenectomy	The tumor recurred at 4 months, and died two weeks later	Baber A et.al (12)
71/female	Dizziness and headache, without abdominal distention, fatigue, fever, weight loss	MRI showed a 45 mm × 38 mm × 40 mm unrepresentative round lump in the spleen	Laparoscopic partial splenectomy	No recurrence or metastasis was found	Chen Y et.al (8)
77/Female	Nonspecific discomfort and exhaustion six weeks prior to admission.	CT scans showed significant splenomegaly with two irregular soft tissue density masses in the spleen	Laparoscopic total splenectomy	Not followed up	Zhao X et.al (13)
59/Female	No significant discomfort	The CT scan indicates a low-density mass in the spleen, measuring 41mm in diameter	Partial splenectomy	Still alive	Nie C et.al (9)
69/Female	No significant discomfort	CT-scan showing a 43×40×39 mm lesion of the inferior pole of the spleen	Splenectomy and caudal pancreatectomy.	Not followed up	Ungureanu IA et.al (14)
68/Female	No significant discomfort	MRI with abdominal venography revealed a well-defined 3.7 × 3.7 cm solid lesion in the lower pole of the spleen	Robotic-assisted diagnostic splenectomy	Not followed up	Eid MK et.al (15)

### Epidemiology and clinical manifestations

EBV^+^ IFDCS is a rare malignancy, accounting for less than 0.4% of all soft tissue sarcomas (16). It is reported more frequently in Asian populations, possibly due to geographic differences in EBV prevalence (17). The age range of affected patients spans from 21 to 84 years, with a median age of 52-56.5 years and a slight female predominance (4, 18). The liver and spleen are the most common sites, comprising 52.6% and 44.7% of cases, respectively (3). Rarely, it may occur in extra-hepatic and extra-splenic sites such as the colon, lung, retroperitoneum, and head and neck (2, 5). Clinical manifestations are nonspecific and largely depend on the tumor’s size and location. Approximately 50%-60% of cases are asymptomatic, discovered incidentally; others present with abdominal discomfort or pain (28.3%), palpable mass (15.1%), or nonspecific systemic symptoms such as fatigue and weight loss (7.5%) (10, 18). Moreover, EBV^+^ IFDCS have been reported with concomitant paraneoplastic pemphigus, characterized clinically by mucocutaneous blistering and erosions, possibly resulting from tumor-induced autoantibodies (5). Laboratory findings are generally nonspecific; some patients exhibit mild anemia, thrombocytopenia, or elevated inflammatory markers (10). Dong Ni Leng et al. reported a case of splenic EBV^+^ IFDCS presenting initially with thrombocytopenia. After splenectomy, the platelet count returned to normal, suggesting that the tumor may influence platelet production or destruction through certain mechanisms (11). Furthermore, elevated serum IgG4 or IgG4^+^ plasma cells have been noted in ~30% of cases, though the significance remains unclear (19, 20). It is noteworthy that although most cases of EBV^+^ IFDCS exhibit slow clinical progression and indolent behavior, they generally have a favorable prognosis following surgical treatment, but a small number of cases present with a more aggressive clinical course. For instance, Alistair Baber and colleagues reported a case of splenic EBV^+^ IFDCS presenting with abdominal pain, rapid deterioration of general condition, a strong inflammatory response, and hypercalcemia (12). The patient experienced recurrence and metastasis within four months after surgery and unfortunately died. Although such highly aggressive progression is relatively rare, it warrants clinical attention.

### Imaging characteristics

Given the rarity of EBV^+^ IFDCS, its imaging characteristics have not yet been clearly defined. In recent years, researchers have gradually begun to explore the features of this entity. A study by Yafang Chen et al. reported that splenic EBV^+^ IFDCS exhibited nodular enhancement during the arterial and portal venous phases on MRI, with a distinctive pattern of reversed enhancement in the delayed phase (8). Another study provided a detailed analysis of the PET/CT imaging characteristics of a case of splenic EBV^+^ IFDCS, revealing the following typical features: (1) frequent hemorrhage and necrosis; (2) persistent moderate enhancement in the tumor parenchyma; (3) capsule-like rim enhancement at the lesion margin; and (4) potentially high standardized uptake value, indicating active glucose metabolism. On non-contrast CT, EBV^+^ IFDCS typically appears as a well-defined solitary mass with heterogeneous density, which is associated with the common presence of hemorrhage, necrosis, and fibrosis within the tumor (13). Contrast-enhanced imaging revealed that the tumor typically exhibited a progressive enhancement pattern: mild heterogeneous enhancement in the arterial phase, with persistent enhancement during the portal venous and delayed phases. This corresponds to the pathological features of the tumor, which include abundant fibrous components and inflammatory cell infiltration (8). A report retrospectively reviewed the CT and MRI imaging features of mesenchymal tumors of splenic origin (21), including EBV^+^ IFDCS, and concluded that although certain characteristics may provide clues for a preliminary diagnosis, definitive diagnosis still requires further confirmation through histopathology and immunohistochemistry. Overall, the imaging features of EBV^+^ IFDCS are nonspecific and vary significantly among patients; therefore, pathological examination remains the key method for definitive diagnosis.

### Pathological features

Macroscopically, EBV^+^ IFDCS appears as a solid mass with clear boundaries, gray-white to yellowish cut surface, and moderate to firm consistency, often with hemorrhagic, necrotic, or cystic areas (3, 4). The tumor size varies considerably, ranging from 1.5 cm to 23 cm, with a mean diameter of approximately 6.5 cm (18, 22). EBV^+^ IFDCS located in the colon often present as polypoid lesions, either pedunculated or sessile, with an intact or eroded mucosal surface (2). Under the microscope, the tumor exhibits spindle to oval-shaped cells with varying degrees of differentiation, admixed with a dense infiltrate of lymphocytes and plasma cells (3). Based on morphological features, EBV^+^ IFDCS can be classified into three subtypes: classic, lymphoma-like, and hemangioma-like. Among these, the classic subtype is the most common, characterized by tumor cells arranged in fascicular or storiform patterns, nuclei with a vesicular appearance and small nucleoli, and a background of moderate lymphoplasmacytic infiltration and vascular proliferation (3). The case reported in our study is considered a classic subtype of EBV^+^ IFDCS. Additionally, approximately 15% of cases may exhibit epithelioid granuloma formation, which increases the difficulty of differentiating it from infectious granulomatous diseases (9, 14). The tumor cells typically display mild to moderate cytologic atypia, with mitotic figures being infrequent—generally fewer than 5 per 10 high-power fields, although focal areas may show marked pleomorphism and hyperchromatic nuclei (4). Necrosis and vascular invasion are observed in approximately 20% of cases and are more commonly seen in recurrent or metastatic lesions (12).

The diagnosis of EBV^+^ IFDCS relies on immunohistochemical confirmation of FDC differentiation in tumor cells. These cells typically express one or more FDC markers, including CD21, CD23, and CD35 (3, 18). Notably, partial loss of antigen expression may occur in tumor cells; therefore, a panel of multiple FDC markers is recommended to enhance diagnostic sensitivity (4). In addition to FDC markers, EBV^+^ IFDCS frequently expresses SMA, PD-L1, and SSTR2a (23, 24).

EBER *in situ* hybridization is essential for the diagnosis of EBV^+^ IFDCS and is considered the gold standard. Nearly all cases show strong nuclear positivity in tumor cells (3). EBV plays a critical role in the pathogenesis of EBV+ IFDCS. Studies have demonstrated that nearly all EBV+ IFDCS cases harbor monoclonal EBV genome integration, suggesting that viral infection occurs prior to clonal expansion of the tumor (17). Similar to EBV-associated gastric carcinoma, EBV^+^ IFDCS typically exhibits a latency type II infection pattern, characterized by the expression of EBNA1, LMP1, and LMP2A, but not EBNA2 (17, 25). EBV may promote tumorigenesis through several mechanisms (17, 25): (1) LMP1 activates the NF-κB and JAK/STAT pathways, promoting cell survival and proliferation;(2) EBV-encoded microRNAs modulate host gene expression, suppress apoptosis, and evade immune surveillance;(3) Viral proteins induce epigenetic changes that silence tumor suppressor genes. In addition, the chronic inflammatory microenvironment induced by EBV infection may promote genomic instability and malignant transformation through the production of reactive oxygen species and pro-inflammatory cytokines (26). Whole-genome and targeted sequencing studies have revealed that approximately 90% of EBV^+^ IFDCS cases harbor alterations in genes regulating the NF-κB signaling pathway, such as TRAF3, CYLD, and NFKBIA, resulting in constitutive NF-κB activation and an inflammatory microenvironment (4, 9). Aberrations in cell cycle regulation and epigenetic control may also be present (9, 12, 24). Of particular note, a recent study reported a G618R mutation in the STAT3 gene in one EBV^+^ IFDCS case, suggesting that activation of the JAK/STAT pathway may contribute to pathogenesis in a subset of patients (27). Additionally, increased MDM2 expression has been observed in approximately 36% of cases, with MDM2 gene amplification detected in some, which may promote tumorigenesis by inhibiting the p53 pathway (24).

There have also been reports describing the ultrastructural features of tumor cells in EBV^+^ IFDCS using electron microscopy. Researchers observed that the tumor cells possess complex dendritic processes connected by desmosomes, with intermediate filaments and lysosome-like granules present in the cytoplasm (28). These features are similar to the ultrastructure of normal follicular dendritic cells, supporting the tumor’s origin from FDCs (1).

### Differential diagnosis

In clinical practice, EBV^+^ IFDCS must be differentiated from a variety of other diseases. The main differential diagnoses include classic follicular dendritic cell sarcoma (FDCS) (9), various types of lymphoma (especially Hodgkin lymphoma and T-cell lymphoma) (9), vascular tumors (3), inflammatory myofibroblastic tumor (14), and certain granulomatous diseases such as tuberculosis and sarcoidosis (9, 29). A definitive diagnosis requires an integrated assessment of clinical presentation, histopathological features, immunohistochemical markers, and EBER *in situ* hybridization results. EBV^+^ IFDCS should be considered in the differential diagnosis when a patient presents with an inflammatory mass in the liver or spleen, or with polypoid lesions in the gastrointestinal tract accompanied by a prominent inflammatory background. If histological examination reveals scattered atypical spindle or oval cells embedded within a dense lymphoplasmacytic background, and these cells are positive for follicular dendritic cell markers such as CD21, CD23, or CD35, there should be a high index of suspicion for EBV^+^ IFDCS. Ultimately, confirmation of EBV infection in tumor cells by EBER *in situ* hybridization remains the key to establishing the diagnosis.

### Treatment and prognosis

Currently, the primary treatment for EBV^+^ IFDCS is surgical resection, aiming for complete removal of the lesion. Xiaokang Ke and Jiahui Hu et al. reported that EBV^+^ IFDCS located in the colon can be treated by endoscopic resection of the polypoid lesion, with no recurrence observed during postoperative follow-up periods ranging from 7 to 12 months (2, 20). In addition, for cases located in the liver or spleen, local lesion resection is typically performed (8, 29), with surgical intervention offering the best chance for cure (10, 17). Retrospective studies have shown that patients who undergo R0 resection have a 5-year progression-free survival rate exceeding 85%, which is significantly higher than that of patients with incomplete resection (9, 18).

There is currently no consensus on the need for adjuvant therapy. In the present case, preoperative imaging suggested a benign lesion, and the tumor was confined to the middle and lower pole of the spleen—consistent with previous reports (8). Therefore, laparoscopic partial splenectomy was performed. For patients with unresectable, recurrent, or metastatic disease, chemotherapy and radiotherapy may be considered as adjunctive treatments, although their efficacy remains uncertain. Alistair Baber et al. reported a case of aggressive EBV^+^ IFDCS in which treatment with gemcitabine was ineffective, and the patient’s condition rapidly deteriorated, leading to death. This suggests that conventional chemotherapy may have limited efficacy in some aggressive cases.

With growing understanding of the molecular pathogenesis of this disease, personalized therapies targeting specific pathways may become available in the future. From a prognostic perspective, EBV^+^ IFDCS generally follows an indolent clinical course and is associated with a better prognosis compared to classic FDCS. One study reported a 3-year progression-free survival rate of 66.67% for EBV+ IFDCS, significantly higher than the 16.67% observed in classic FDCS (9). However, some cases suggest the existence of highly aggressive subtypes with rapid progression and poor prognosis, potentially associated with specific genetic mutations such as CDKN2A or NF1 (12). These patients may require more aggressive treatment strategies and closer monitoring.Postoperative follow-up is critical. It is recommended that imaging evaluations be performed every 3–6 months during the first 2 years, with longer intervals thereafter as appropriate (9).

## Conclusion

EBV^+^ IFDCS is a rare tumor with distinct biological features. Accurate diagnosis requires high clinical and pathological suspicion. Multidisciplinary collaboration and individualized treatment are essential to improve outcomes. Advances in molecular biology and understanding of EBV-driven oncogenesis may pave the way for improved diagnostics and targeted therapies in the future.

## Data Availability

The raw data supporting the conclusions of this article will be made available by the authors, without undue reservation.

## References

[1] Chan JK Fletcher CD Nayler SJ Cooper K. Follicular dendritic cell sarcoma. Clinicopathologic analysis of 17 cases suggesting a Malignant potential higher than currently recognized. Cancer. (1997) 79:294–313. doi: 10.1002/(SICI)1097-0142(19970115)79:2<294::AID-CNCR13>3.0.CO;2-W, PMID: 9010103

[2] Ke X He H Zhang Q Yuan J Ao Q. Epstein-Barr virus-positive inflammatory follicular dendritic cell sarcoma presenting as a solitary colonic mass: two rare cases and a literature review. Histopathology. (2020) 77:832–40. doi: 10.1111/his.14169, PMID: 32506505

[3] Li Y Yang X Tao L Zeng W Zuo M Li S. Challenges in the diagnosis of epstein-barr virus-positive inflammatory follicular dendritic cell sarcoma: extremely wide morphologic spectrum and immunophenotype. Am J Surg Pathol. (2023) 47:476–89. doi: 10.1097/PAS.0000000000002011, PMID: 36574358

[4] Facchetti F Simbeni M Lorenzi L. Follicular dendritic cell sarcoma. Pathologica. (2021) 113:316–29. doi: 10.32074/1591-951X-331, PMID: 34837090 PMC8720404

[5] Jiang XN Zhang Y Xue T Chen JY Chan ACL Cheuk W. New clinicopathologic scenarios of EBV+ Inflammatory follicular dendritic cell sarcoma: report of 9 extrahepatosplenic cases. Am J Surg Pathol. (2021) 45:765–72. doi: 10.1097/PAS.0000000000001632, PMID: 33264138

[6] Chong J Jones P Spelman D Leder K Cheng AC. Overwhelming post-splenectomy sepsis in patients with asplenia and hyposplenia: a retrospective cohort study. Epidemiol Infect. (2017) 145:397–400. doi: 10.1017/S0950268816002405, PMID: 27776576 PMC9507625

[7] Kinjo N Kawanaka H Akahoshi T Tomikawa M Yamashita N Konishi K. Risk factors for portal venous thrombosis after splenectomy in patients with cirrhosis and portal hypertension. Br J Surg. (2010) 97:910–6. doi: 10.1002/bjs.7002, PMID: 20474001

[8] Chen Y Yu X. A case report: spleen Epstein-Barr virus-positive inflammatory follicular dendritic cell sarcoma. J Gastrointestinal Oncol. (2024) 15:2706–11. doi: 10.21037/jgo-24-483, PMID: 39816035 PMC11732363

[9] Nie C Xie X Li H Li Y Chen Z Li Y. Epstein-Barr virus-positive inflammatory follicular dendritic cell sarcoma with significant granuloma: case report and literature review. Diagn Pathol. (2024) 19:34. doi: 10.1186/s13000-024-01457-6, PMID: 38365739 PMC10870656

[10] Jin J Zhu X Wan Y Shi Y. Epstein-barr virus (EBV)-positive inflammatory pseudotumor-like follicular dendritic cell sarcoma (IPT-like FDCS) presenting as thrombocytopenia: A case report and literature review. Heliyon. (2024) 10:e32997. doi: 10.1016/j.heliyon.2024.e32997, PMID: 38994118 PMC11238001

[11] Leng DN Yu KJ Wang J. Inflammatory pseudotumor-like follicular dendritic cell sarcoma with first clinical manifestation of thrombocytopenia: A case report. Medicine. (2022) 101:e32528. doi: 10.1097/MD.0000000000032528, PMID: 36596072 PMC9803453

[12] Baber A Legendre P Palmic P Lupo-Mansuet A Burroni B Azoulay C. EBV-positive inflammatory follicular dendritic cell sarcoma of the spleen: report of an aggressive form with molecular characterization. Int J Surg Pathol. (2024) 32:150–4. doi: 10.1177/10668969231168345, PMID: 37157817

[13] Zhao X Gai L Wang L Xu L. Imaging findings of Epstein-Barr Virus-positive inflammatory follicular dendritic cell sarcoma of spleen: A case report. Technol Health Care: Off J Eur Soc Eng Med. (2024) 32:437–45. doi: 10.3233/THC-248038, PMID: 38759066 PMC11191520

[14] Ungureanu IA Lupinacci RM Parrens M Emile JF. Granulomatous splenic mass with necrosis revealing an EBV-positive inflammatory follicular dendritic cell sarcoma. J Surg Case Rep. (2022) 5:rjac034. doi: 10.1093/jscr/rjac034, PMID: 35531436 PMC9071999

[15] Eid MK AlQaqaa AS Mohammed IJ Shahait AD. A rare case of an EBV-positive inflammatory follicular dendritic cell tumor of the spleen. J Surg Case Rep. (2024) 9:rjae600. doi: 10.1093/jscr/rjae600, PMID: 39324102 PMC11421992

[16] Deng S Gao J. Inflammatory pseudotumor-like follicular dendritic cell sarcoma: a rare presentation of a hepatic mass. Int J Clin Exp Pathol. (2019) 12:3149–55., PMID: 31934158 PMC6949706

[17] Abe K Kitago M Matsuda S Shinoda M Yagi H Abe Y. Epstein-Barr virus-associated inflammatory pseudotumor variant of follicular dendritic cell sarcoma of the liver: a case report and review of the literature. Surg Case Rep. (2022) 8:220. doi: 10.1186/s40792-022-01572-w, PMID: 36484868 PMC9733763

[18] Ge R Liu C Yin X Chen J Zhou X Huang C. Clinicopathologic characteristics of inflammatory pseudotumor-like follicular dendritic cell sarcoma. Int J Clin Exp Pathol. (2014) 7:2421–9., PMID: 24966952 PMC4069939

[19] Choe JY Go H Jeon YK Yun JY Kim YA Kim HJ. Inflammatory pseudotumor-like follicular dendritic cell sarcoma of the spleen: a report of six cases with increased IgG4-positive plasma cells. Pathol Int. (2013) 63:245–51. doi: 10.1111/pin.12057, PMID: 23714251

[20] Hu J Huang D Xu C Chen Y Ma H Shen Z. Epstein-barr virus-positive inflammatory follicular dendritic cell sarcoma presenting as a colonic polyp: report of a case with a literature review. Med (Kaunas Lithuania). (2023) 59. doi: 10.3390/medicina59071341, PMID: 37512154 PMC10385426

[21] Prasad AS Chua SS Ramani NS Shiralkar KG Shanbhogue KP Surabhi VR. Stroma-derived neoplasms and pseudoneoplastic lesions of the spleen: a select review of pathologic and CT/MRI findings. Abdominal Radiol (New York). (2025) 50:480–95. doi: 10.1007/s00261-024-04461-y, PMID: 38937338

[22] Lee CH Yen YS Shan YS Lin PW. Follicular dendritic cell tumor in liver: A case report and collective review. Gastroenterol Res. (2010) 3:139–43. doi: 10.4021/gr2010.05.206w, PMID: 27942292 PMC5139768

[23] Pardasani M Rajakannu M Vij M Rajalingam R Rela M. Aggressive variant of hepatic epstein-barr virus-associated inflammatory pseudotumor-like follicular dendritic cell sarcoma with PD-L1 and SSTR2a expression. Diagnostics (Basel Switzerland). (2023) 13. doi: 10.3390/diagnostics13182916, PMID: 37761283 PMC10529831

[24] Agaimy A Michal M Hadravsky L Michal M. Follicular dendritic cell sarcoma: clinicopathologic study of 15 cases with emphasis on novel expression of MDM2, somatostatin receptor 2A, and PD-L1. Ann Diagn Pathol. (2016) 23:21–8. doi: 10.1016/j.anndiagpath.2016.05.003, PMID: 27402219

[25] Chen S Deng Y Huang C Xie X Long Z Lao S. BSRF1 modulates IFN-β-mediated antiviral responses by inhibiting NF-κB activity via an IKK-dependent mechanism in Epstein-Barr virus infection. Int J Biol Macromol. (2025) 306:141600. doi: 10.1016/j.ijbiomac.2025.141600, PMID: 40024405

[26] Patrad E Khalighfard S Amiriani T Khori V Alizadeh AM. Molecular mechanisms underlying the action of carcinogens in gastric cancer with a glimpse into targeted therapy. Cell Oncol (Dordrecht Netherlands). (2022) 45:1073–117. doi: 10.1007/s13402-022-00715-3, PMID: 36149600 PMC12978125

[27] Ramsey MC Sabatini PJB Watson G Chawla T Ko M Sakhdari A. Case Report: Identification of a novel STAT3 mutation in EBV-positive inflammatory follicular dendritic cell sarcoma. Front Oncol. (2023) 13:1266897. doi: 10.3389/fonc.2023.1266897, PMID: 37965457 PMC10640977

[28] Perez-Ordoñez B Rosai J. Follicular dendritic cell tumor: review of the entity. Semin Diagn Pathol. (1998) 15:144–54., PMID: 9606805

[29] Shen HGD Zhang Y Leow WQ. Uncommon granulomatous manifestation in Epstein-Barr virus-positive follicular dendritic cell sarcoma: a case report. J Pathol Trans Med. (2025) 59:133–8. doi: 10.4132/jptm.2024.09.27, PMID: 39473040 PMC12010868

